# Activation of human macrophages by human corneal allogen *in vitro*

**DOI:** 10.1371/journal.pone.0194855

**Published:** 2018-04-04

**Authors:** Paola Kammrath Betancor, Antonia Hildebrand, Daniel Böhringer, Florian Emmerich, Günther Schlunck, Thomas Reinhard, Thabo Lapp

**Affiliations:** 1 Eye Center, Medical Center, Faculty of Medicine, University of Freiburg, Freiburg, Germany; 2 Institute for Transfusion Medicine and Gene Therapy, Medical Center, Faculty of Medicine, University of Freiburg, Freiburg, Germany; INSERM, FRANCE

## Abstract

**Purpose:**

To study distinct aspects of human monocyte-derived macrophage (MDM) activation by human corneal tissue as a possible initial stage in human corneal allograft rejection.

**Methods:**

Human monocytes were isolated from peripheral blood mononuclear cells (PBMC) and differentiated into MDM. Human corneas with or without endothelium were fragmented using a standardized protocol. MDM were stimulated with human corneal fragments, corneal fragment supernatant, lipopolysaccharide (LPS) or interferon-gamma (IFNγ), and expression profiles for 34 cytokines were determined in MDM-conditioned media using a Luminex bead-based multiplex assay. Data from clinical aqueous humour samples served for comparison and validation. To assess cell recruitment, immunogenicity of corneal endothelial cells (CEC), monocyte survival and differentiation, we applied transwell migration assays, cell viability assays and fluorescence-activated cell sorting, respectively.

**Results:**

Corneal fragments induced MDM to release distinct cytokines into the medium. Media thus conditioned *in vitro* by stimulated MDM shared cytokine patterns, namely MCP-1, MIP-1α and MIP-1β, with human aqueous humor samples obtained in human corneal allograft rejection. The presence of CEC in tissue fragments used for MDM stimulation attenuated the upregulation of distinct pro-inflammatory chemokines, like MCP-3 and IL-8, reduced the monocyte survival time, and diminished monocyte-to-macrophage differentiation induced by conditioned media. Distinct anti-inflammatory cytokines, like IL-4 and IL-13, were upregulated in the presence of corneal endothelium. Cornea fragment-stimulated MDMs induced recruitment of monocytes from a PBMC pool in a transwell migration model, modulated immune cell viability and promoted further immune cell recruitment and differentiation.

**Conclusions:**

Human macrophages respond to allogenic corneal tissue and generate an inflammatory milieu. This can drive further recruitment of immunocompetent cells and modulate cell survival and differentiation of the cells recruited. These observations are consistent with the hypothesis that macrophages play a significant role in the initiation of corneal transplant rejection. Our data also indicate that distinct aspects of early human corneal transplant rejection can be modelled *in vitro*.

## Introduction

Corneal transplantation is one of the most common tissue transplantations worldwide [1]. The success of the procedure is limited by immune-mediated transplant rejection: up to 25% of the grafts fail due to corneal graft rejection within the first five years, rejection rates being even higher in high-risk cohorts [2]. An immunological host response against transplanted tissue is the leading cause of corneal graft failure characterized by corneal endothelial cell loss and subsequent graft opacification.

The role of the adaptive immune system in corneal graft rejection has been studied extensively, but less is known about the influence of innate immune cells [3,4]. Murine keratoplasty models have revealed T_regs_ to be necessary for allograft survival [5]. Corneal grafts are able to induce the generation of donor-specific T_regs_, which decrease the immune response and improve graft survival [6,7]. Other T cell populations, such as CD4^+^ T cells have been shown to be involved in the pathogenesis of corneal graft rejection. CD4-deficient mice as well as rodents treated with anti-CD4 antibodies showed a reduced corneal graft rejection rate. However, graft rejection can still occur in the absence of CD4^+^ T cells [8].

Clinical studies suggest a significant contribution of the innate immune system to the initiation of corneal graft rejection: macrophages and monocytes were shown to be the predominant cell types in human anterior chamber samples from patients with acute corneal graft rejection [9–11]. Along these lines, macrophage depletion was shown to prolong graft survival in a rat keratoplasty model [12].

The initial steps of corneal graft rejection leading to an inflammatory aqueous humour milieu, cell recruitment into the anterior chamber, and subsequent death of CEC are still poorly characterized. The data currently available allow several conclusions: antigen detection within the allograft is promoted by antigen-presenting cells (APC) [13]. Graft antigen is recognized either in a direct pathway mediated by donor-APCs or in an indirect pathway, in which the recipient’s APCs migrate into the allograft [14]. Following (direct or indirect) APC activation, various cytokines and chemokines as well as recruited monocytes have been identified in the anterior chamber [11]. The common final path of these anterior chamber alterations is the loss of CEC which is likely triggered by cytokines as well as the recruited immune cells. Unfortunately, the cellular mechanisms in the initial phase of corneal allograft rejection remain elusive, and a definite role of macrophages still needs to be determined. In particular, there is currently no conclusive explanation for how these cells are activated and recruited. The question arises, whether macrophages are able to directly recognize corneal allogen and mount a distinct response.

**Fig 1:**
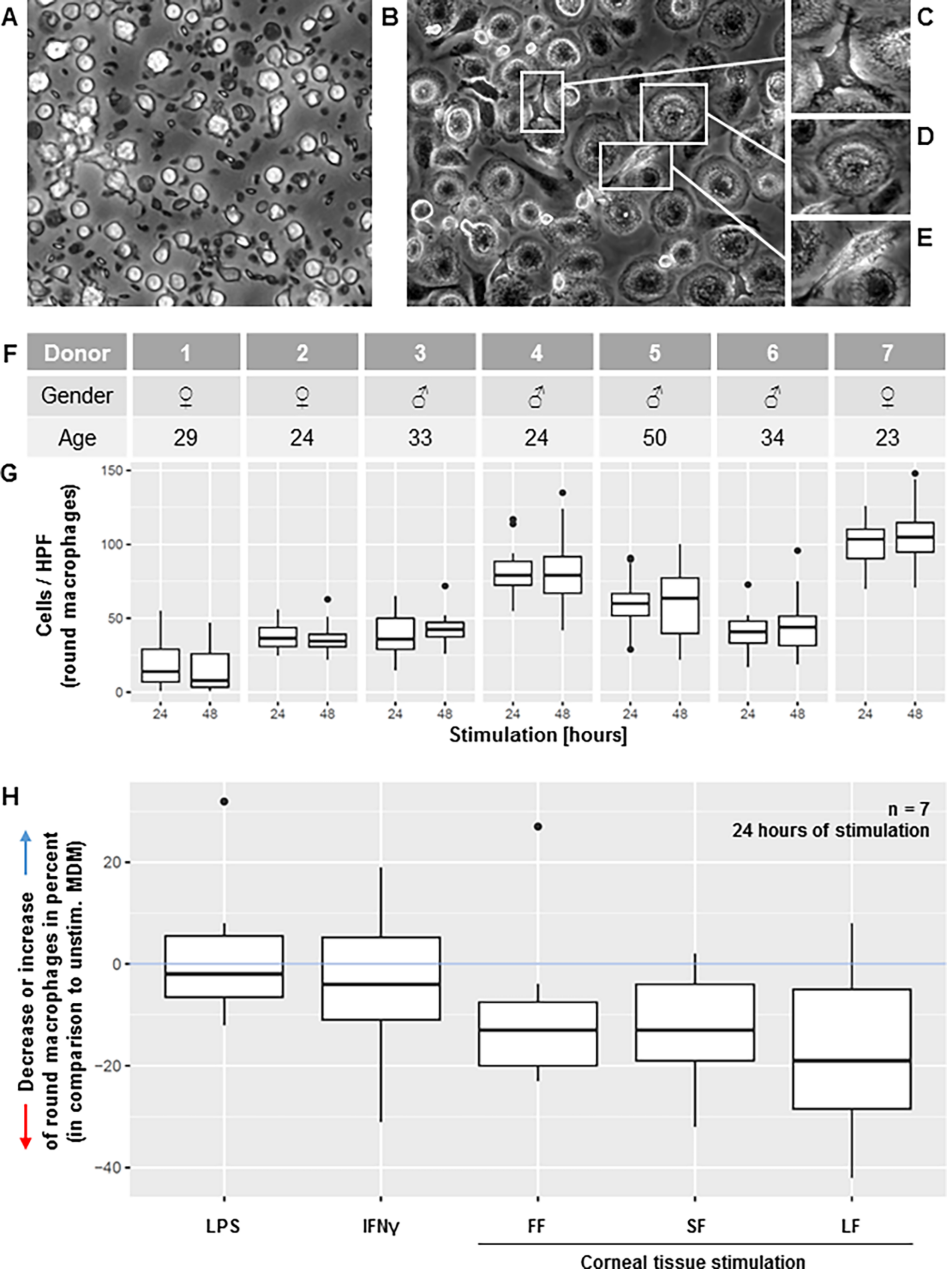
Macrophage morphology analysis. (A) Unstimulated isolated monocytes after 24 hours of incubation, partly apoptotic. (B) Unstimulated MDM, i.e. monocytes stimulated with M-CSF, after 24 hours of incubation; different phenotypes of macrophages were identified as dendritiform (C), round (D), and elongated (E). (F, G) Individual donors revealed distinct distribution patterns for all three macrophage phenotypes (only round phenotype presented, median of all stimuli at 24 and 48 hours). (H) Increase or decrease of round macrophages in percent in different stimulation conditions (lipopolysaccharide [LPS], interferon gamma [IFNγ]), fragment-free supernatant fluid phase [FF], small [SF], and large corneal fragments [LF]) relative change to cell counts in unstimulated samples at 24 hours of incubation (pooled data from 7 individual experiments with individual donors as in Fig 1F).

**Fig 2:**
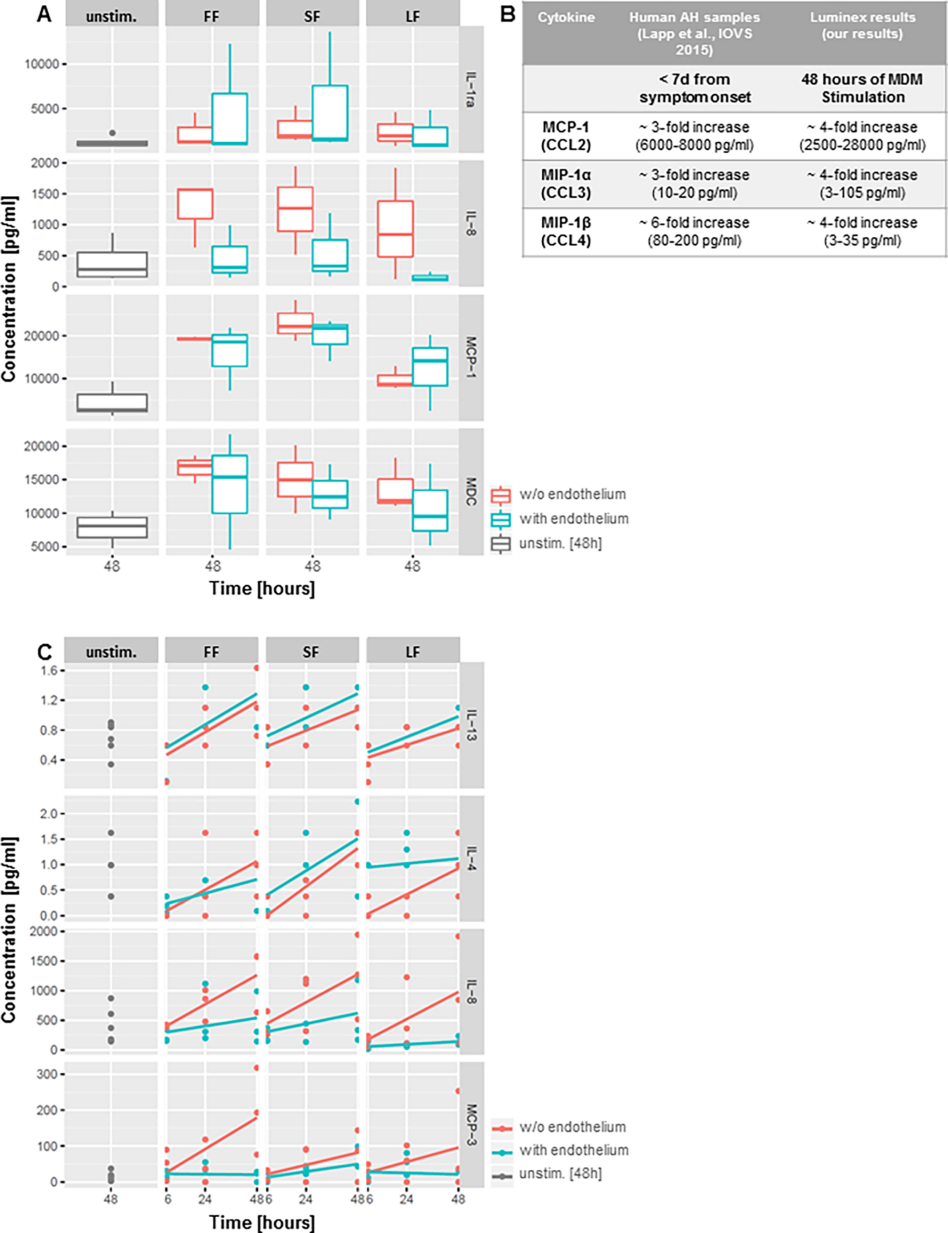
Cytokine patterns. (A) Examples of cytokine levels produced by MDM after stimulation with fragment-free supernatant fluid phase [FF], small [SF], and large corneal fragments [LF] with (blue) or without endothelium (red) or control media after 48 hours. Unstimulated MDM (supernatant collected after 48h) and untreated corneal tissue (without MDM-co-incubation) served as negative controls; MDM stimulated with LPS or IFNγ served as positive controls (only unstimulated control shown). (B) Cytokines in aqueous humor (AH) samples from patients during acute corneal graft rejection (duration of symptoms < 7 days; fold increase compared to AH samples from patients undergoing cataract surgery w/o history of other corneal pathologies), and in MDM supernatant after 48 hours of stimulation *in vitro* (fold increase related to six versus 48 hours of stimulation). (C) Time course of individual cytokine concentrations produced by MDM incubated with FF, SF, and LF; concentrations given for six, 24 and 48 hours of stimulation. The presence (blue) or absence (red) of corneal endothelium led to an increase of anti-inflammatory chemokines (IL-4 and IL-13) when endothelium was present during MDM-stimulation; vice versa to an increase of inflammatory / chemotactic cytokines (IL-8 and MCP-1) when corneal stroma without endothelium was used for MDM-stimulation.

**Fig 3:**
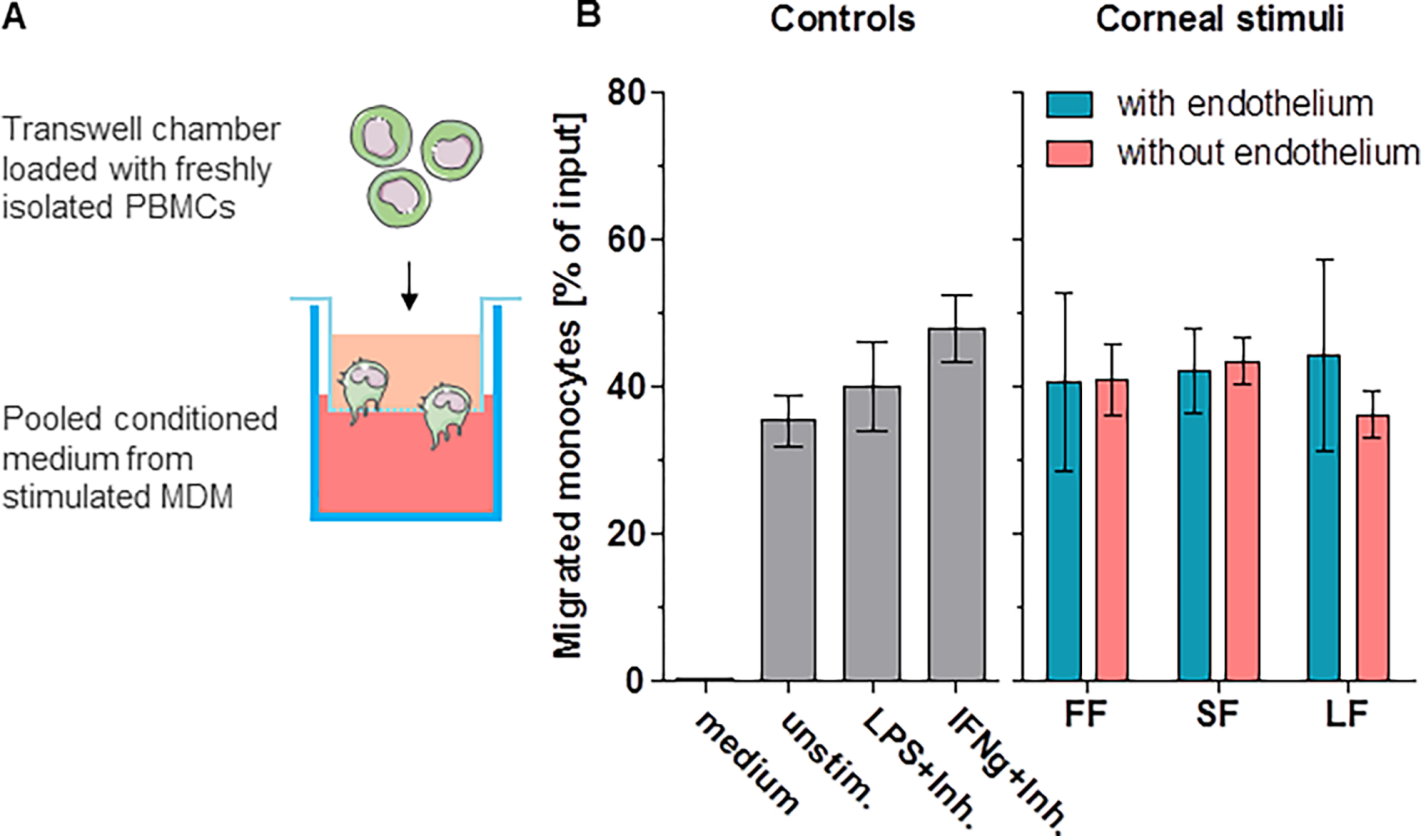
Migration analysis. (A) Experimental setup for migration analysis. Wells were loaded with pooled supernatant from stimulated MDM. Freshly isolated PBMC were loaded into the upper chamber of transwell inserts. After three hours of migration, cells were identified and counted using FACS. (B) Results of the migration assays. Depicted is the percentage of migrated cells in relation to the total input. Corneal tissue used for MDM stimulation induced up-regulation of various cytokines (see Fig 1); supernatant from MDM with these cytokines induces monocyte recruitment.

**Fig 4:**
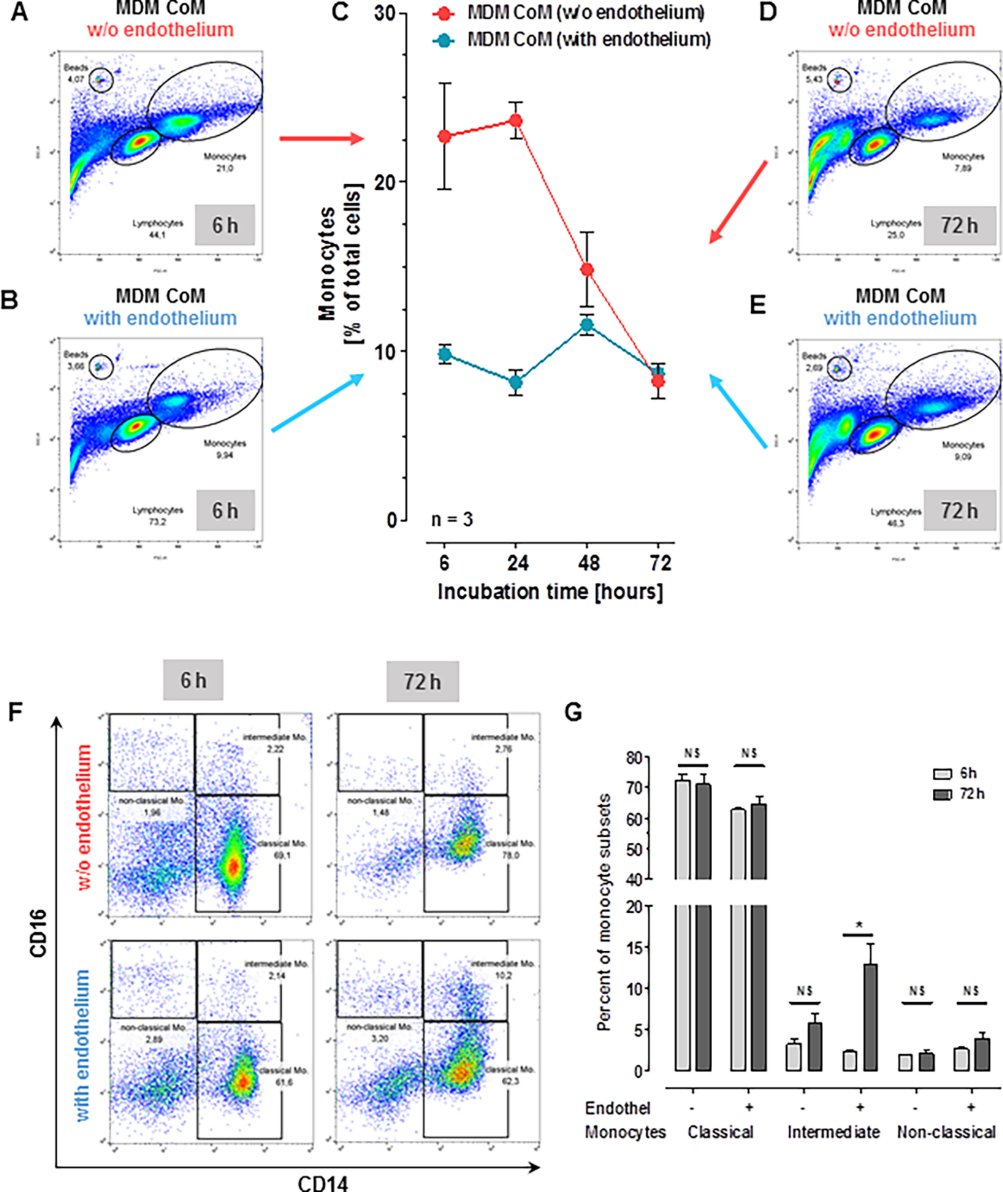
Monocyte fraction persistence. (A—E) For analysis, freshly isolated monocytes were incubated in pooled supernatant from MDM stimulated with corneal tissue; monocytes were identified using flow cytometry (depicted for six (A and B) and 72 hours (D and E) of incubation). The monocyte fraction was determined based on the percentage of gated cells shown in flow cytometry plots. FACS was performed after six, 24, 48 and 72 hours of incubation (Fig 4C). Counting beads were used to ensure consistent cell counts in all samples. The presence (blue) or absence (red) of corneal endothelium (CEC) during the initial MDM stimulation is indicated. In the absence of CEC twice as many monocytes can be found after six hours of incubation. Gated monocytes (as in A, B, D, and E) were further sub-differentiated into classical (CD14++CD16−), intermediate (CD14++CD16+), and non-classical (CD14+CD16++) monocytes (Fig 4F). Monocytes incubated with supernatant from MDM stimulated with endothelium showed a statistically significant increase in intermediate monocytes (Fig 4F and G; * p<0.05).

**Fig 5:**
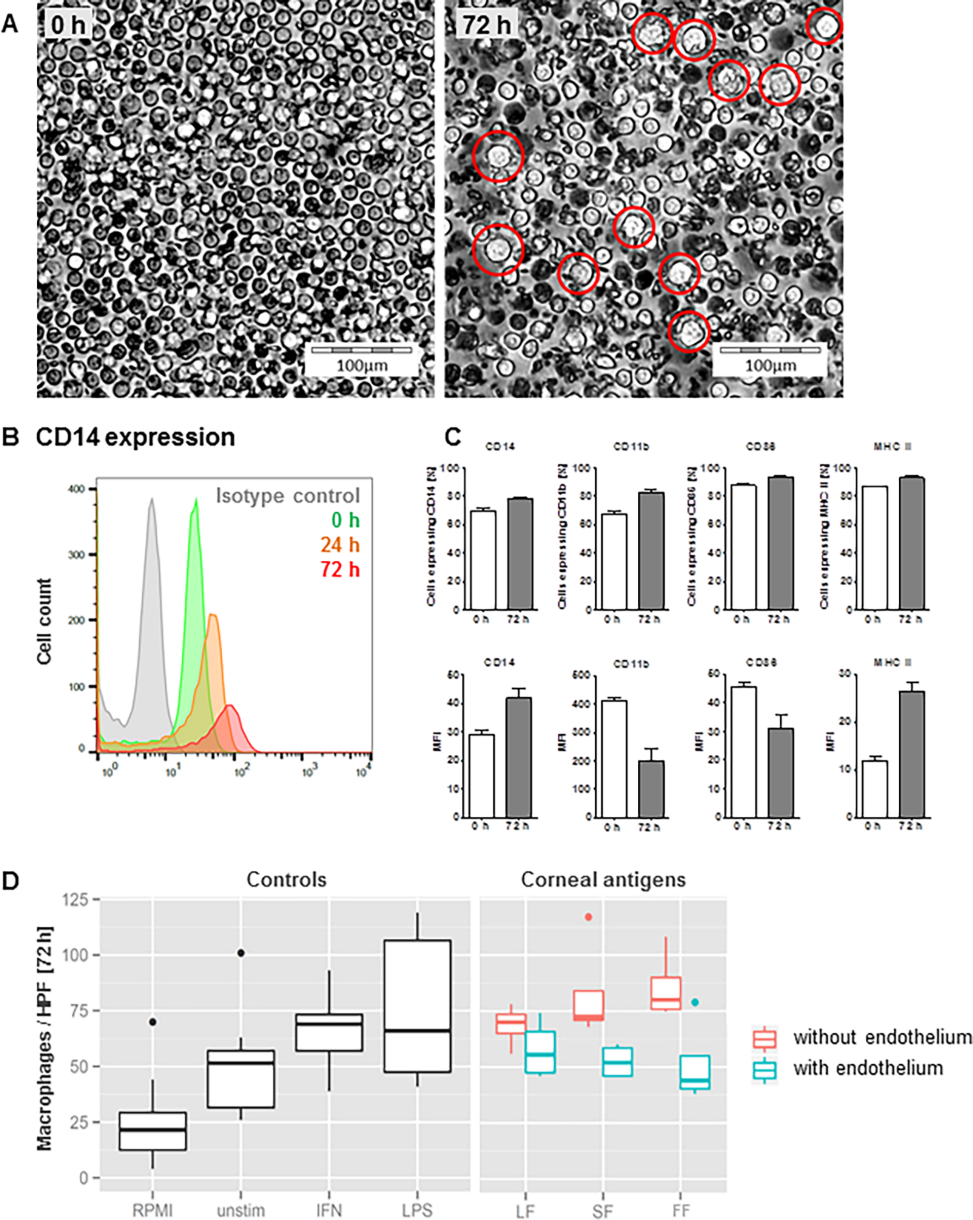
Monocyte-to-macrophage differentiation. (A) Freshly isolated monocytes (0 h) and monocytes after 72 hours of incubation in supernatant of MDM stimulated with corneal antigen (exemplary picture of a setup stimulated with CECs). The red circles indicate monocytes with a macrophage-like morphology. (B, C) Expression of e.g. CD14 (B) and further markers (C) in freshly isolated monocytes and monocytes with a macrophage-like morphology after 24 and 72 hours of (as in A) incubation in MDM-conditioned medium. (D) Counts of cells with a macrophage-like morphology per high power field after 72 hours of incubation.

Therefore, we set up a human *in vitro* model to study responses of macrophages and monocytes to human corneal material and investigate their possible role in the early stages of corneal allograft rejection. The experiments were planned to address different steps of antigen-processing during the initial cascade of graft rejection: (1) corneal allogen recognition, antigen uptake and processing by macrophages (Fig 1); (2) subsequent macrophage activation as determined by measuring their cytokine response (Fig 2) (3) potential recruitment of further cells (notably monocytes) (Fig 3), and finally (4) monocyte apoptosis in the absence of stimulus (Fig 4) or further differentiation into APCs (Fig 5).

For modelling the anterior chamber milieu during graft rejection we used supernatant from monocyte-derived macrophages (MDM) which had been stimulated with corneal antigen. This supernatant contains cytokines that MDM had secreted in response (the supernatant is referred to as “MDM-conditioned medium” (CoM)). MDM were differentiated from healthy donors according to previously published methods [15] and stimulated with dissociated cornea–with or without endothelium–provided by the Lions Cornea Bank Baden-Württemberg (Freiburg, Germany).

MDM incubation with corneal antigen induced a smaller fraction of round macrophages than incubation either with LPS, IFNγ, or control medium. This corneal antigen-related attenuation of round macrophages was independent of the presence of corneal endothelium in the antigenic material. All MDM-conditioned media induced similar monocyte recruitment from PBMCs in a transwell migration assay, demonstrating that MDM in culture produce chemokines even without adequate stimulation. In contrast, the presence of CEC in the corneal antigen preparation was associated with attenuated upregulation of distinct pro-inflammatory chemokines in MDM supernatants, reduced monocyte fractions, and diminished monocyte-to-macrophage differentiation.

This *in vitro* approach allows to characterize cellular responses in a well-defined setup and may serve as a valuable complement to current animal models.

## Materials and methods

### Ethics

Ethics approval was granted by the local Ethics Committee (Research Ethics Committee of University of Freiburg; Germany; reference number: 572/14; project title “In vitro stimulation of human APCs with allogenic corneal tissue”). Written informed consent was obtained from all volunteers involved in this study. This study adheres to the tenets of the Declaration of Helsinki.

### Human blood sample processing and preparation of monocyte-derived macrophages (MDM)

Human blood samples were obtained from healthy donors (16 donors, 5 females and 11 males, aged 23 to 50 years with a median age of 30 years). Heparinized blood was centrifuged using a density-gradient (Pancoll Human, PAN Biotech, Aidenbach, Germany). The contained peripheral blood mononuclear cells (PBMC) were retrieved and washed using Hanks buffered saline solution (HBSS) (Gibco, UK). PBMCs were re-suspended in RPMI (Sigma, Saint Louis, USA) with 5% AB human serum (Sigma) and evenly seeded into six-well plates. After two hours of incubation at 37°C with 5% CO_2,_ the remaining non-adherent cells were removed by gently washing the plates with HBSS three times. The adherent monocytes were then incubated for three days in RPMI with 10% autologous human serum and 20 ng/ml macrophage-colony stimulating factor (M-CSF) (R&D Systems, Minneapolis, USA). The resident cells were then again washed with HBSS and incubated for three days in RPMI with 10% autologous serum until the differentiation to MDM was completed [15].

### Disintegration of human corneal tissue and MDM stimulation

To assess the activation of MDM in response to corneal fragments of different size, a standardized protocol was established to shred human corneal tissue. Human corneas were received from the Lions Cornea Bank Baden-Württemberg (Freiburg, Germany) after obtaining informed consent from next of kin. Corneas with endothelium not suitable for transplantation due to a reduced corneal endothelial cell count (endothelial cell count <2000 cells/m^2^, but above 1500 cells/mm^2^) were used for experiments. Corneal tissue without endothelium was derived from donor cornea residues following Descemet’s membrane endothelial keratoplasty (DMEK) implant preparation. Corneas from donors presenting any transmissible diseases were also excluded from the experiments. All corneas were de-epithelialized with a hockey knife and dissected using an 8.0 mm diameter trephine. The corneal tissue was shredded manually with a scalpel and then disintegrated in a tissue lyser for 20 min at 50 Hz (TissueLyser LT, QIAGEN, Hilden, Germany) with stainless steel beads (7mm, Qiagen GmbH, Hilden, Germany). The resulting fragments were separated into small (SF) and large fragments (LF) by subsequent sedimentation steps. SF and LF as well as the fragment-free supernatant fluid phase (FF) were used to stimulate the MDM for various time-intervals (up to 48 hours). Lipopolysaccharide (LPS, Sigma, [100 ng/ml]) and IFNγ (Pepro Tech, New Jersey, USA; [10 ng/ml]) were used as positive controls. MDM stimulation using LPS (six hours) or IFNγ (24 hours) was performed according to a standardized protocol [11,15]. During the stimulation, MDM were incubated at 37°C and 5% CO_2._

### Morphology analysis

MDM morphology was evaluated in all six conditions (unstimulated, LPS, IFNγ, FF, SF and LF) after 24 and 48 hours. As previously described, cells were classified into three different phenotypes (round, elongated, and dendritiform) [16]. Four images of each well were taken at a magnification of 400x in areas of comparable cell density (central as well as peripheral). Unstimulated macrophages were used for reference and the relative decrease or increase in round macrophage count was calculated for the five stimuli in percent. The cells were analyzed and counted with custom software (“Cell Finder”) using the R-platform [17,18]. Cell counts at 24 and 48 hours were compared between individual donors; the effect of different stimuli on MDM morphology was compared with unstimulated MDM.

### Cytokine detection (LUMINEX)

The conditioned media from six individual experiments (three with and three without endothelium) were collected and analysed using a Luminex Assay (HCYTOMAG-60K; EMD Millipore Corporation, Massachusetts, USA) following morphology evaluation. Each sample was tested for 34 cytokines. The conditioned media from the cornea-stimulated samples–with and without endothelium–were sampled after six, 24 and 48 hours of stimulation. The conditioned media of the positive controls, stimulated with LPS (six hours stimulation) or IFNγ (24 hours stimulation), were gathered respectively. For negative controls we used medium conditioned for 48 hours by unstimulated MDM as well as lysates from corneas not incubated with MDM. Luminex assays were validated using additional internal quality controls. Cytokines outside of the detection range were excluded from further analysis.

### Migration assay

Pooled conditioned media from eight individual experiments were used for migration analysis. 500 μl medium conditioned by stimulated MDM (experimental setup as above) were pooled. To exclude LPS- or IFNγ- mediated effects in positive control media, both stimuli were blocked specifically as previously described [11]: 10 mg/ml of Polymyxin B (Sigma) or 500 μg/ml of anti-IFNγ antibody AB9657 (Abcam, Cambridge, UK) were added to inhibit LPS or IFNγ, respectively. 100 μl of freshly isolated PBMC (5x10^6^ cells/ml) were loaded into the upper chamber of a transwell insert (Corning Nr. 3421, 6.5 mm insert, 5.0 μm pore size; Costar, NY, USA). After three hours of incubation cells were counted and analyzed using FACS. A FACScalibur flow cytometer (Becton Dickinson, CA, USA) and FlowJo analysis software (Version 10, FlowJo, OR, USA) were used. Cells were labeled with anti-CD14-FITC (Clone RPA-T4; BD Pharmingen, Heidelberg, Germany) and anti-CD16-PE (Clone 3G8; BioLegend, CA, USA) antibodies. Flow-Check polystyrol fluorospheres (Beckman Coulter, CA, USA) were used to standardize cell counting. Using light scattering properties and CD14/CD16 signals, monocytes and lymphocytes were identified and monocytes sub-classified into classical (CD14^high^CD16^low^), intermediate (CD14^high^CD16^high^), and non-classical (CD14^low^CD16^high^) monocytes. The percentage of cells migrating through transwell membranes was calculated in proportion to the total PBMC input. The migration effects of the three different corneal stimuli were compared. RPMI-medium and medium conditioned by unstimulated MDM served as negative controls and inhibitor-pre-treated LPS- and IFNγ- stimulated MDM supernatant served as positive controls.

### Monocyte fraction persistence analysis and monocyte-to-macrophage differentiation

Monocytes were freshly isolated from human PBMC to evaluate the effect of MDM-conditioned media (i.e. the supernatants of MDM after stimulation with LPS, IFNγ, corneal material or vehicle control). A pan-monocyte-Isolation-Kit (MACS Miltenyi Biotec, Bergisch Gladbach, Germany) was used to retrieve monocytes from freshly isolated PBMCs. Purity > 95% of isolated monocytes was confirmed by FACS. 500 μl of pooled MDM-conditioned medium was loaded into wells. LPS and IFNγ were inhibited as above. 100 μl of freshly isolated monocytes (5x10^6^ cells/ml) in RPMI were added to the conditioned medium. Monocytes and remaining lymphocytes were identified in the FSC/SSC FACS plot (Fig 4A, 4B, 4D and 4E). Monocytes were further sub-differentiated into classical (CD14^++^CD16^−^), intermediate (CD14^++^CD16^+^), and non-classical (CD14^+^CD16^++^) using CD14- and CD16-specific antibodies (as mentioned above; Fig 4F).

To monitor differentiation, cells were harvested and sorted as described above after six, 24, 48 and 72 hours of incubation. Monocytes were again classified as classical, intermediate or non-classical via FACS. Residential cells were characterized after 72 hours of incubation using antibodies against CD14, CD11b, CD86, and MHCII to identify a possible monocyte-to-macrophage differentiation. After 72 hours of incubation, cells were photographed and counted by macrophage-like differentiation patterns using the “Cell Finder” software.

### Data presentation and statistical analysis

#### Cell morphology

For the morphological cell analysis we used custom made in-house software based on the “R”-Platform (package “EBImage”) [17]. The data were plotted as box and whiskers plots for descriptive assessment (package “ggplot2”). Tukey's post hoc test was used to compare inter-individual differences in morphology (Fig 1G, package “multcomp”). To assess a possible effect of different stimuli on macrophage morphology patterns, unstimulated MDM were set as reference. The increase or decrease in round macrophages of the other stimuli was calculated in percent compared to round macrophages in the unstimulated sample. Data of seven individual experiments were merged and visualized as box and whiskers plots (Fig 1H).

#### Cytokine patterns

We plotted the influence of incubation time (six vs. 48 hours of stimulation; Fig 2A) and presence of endothelium on cytokine production, respectively (Fig 2C). A multifactorial linear regression model (function “lm”, base R) was fitted for statistical inference. The p-values were corrected for multiple testing using the Bonferroni method. The p-values are indicated in the S1 Table.

#### Cell migration

Results of the migration experiments were plotted with GraphPad Prism software version 5 (GraphPad Software, Inc., La Jolla, CA, USA). Data are presented as percent (%) of total input; a paired t-test was used to compare the samples prior and after removal of the endothelium (Fig 3B).

#### Monocyte fraction persistence

We used FlowJo (version 10, Ashland, Oregon) for the FACS analysis. Fig 4A and 4B depict the monocytes and lymphocytes in the FSC/SSC FACS plot at 6 hours; Fig 4D and 4E the same cells at 72 hours. We analyzed the cell counts descriptively by means of a line plot of the means and standard deviations versus incubation time (Fig 4C). We used GraphPad Prism (version as mentioned above) for plotting the data from the FACS analysis. The monocyte fraction (Fig 4F) was sub-differentiated using CD14 and CD16 antibodies as described above; the different monocyte sub-populations were plotted by means of a line plot comparing percent of monocytes at 6 h versus 72h. Statistical calculations were performed using a t-test.

#### Macrophage differentiation

We analyzed the expression intensity of our set of markers (see Fig 5B and 5C) at different time points. Fig 5B shows exemplary the histogram of average CD14 expression per monocyte. This figure was again produced by FlowJo (as mentioned above). The aggregated FACS analyses (mean and standard deviation) are presented in Fig 5C. This figure was done with GraphPad Prism (as mentioned above). We compared the compartments under investigation with respect to the macrophage numbers descriptively using box and whiskers plots (Fig 5D). These had been done using R (package “ggplot2”).

## Results

### MDM response to corneal tissue preparations

#### MDM morphology

Without stimulation, isolated monocytes undergo cell death (Fig 1A). If these cells are supplemented with protein and stimulated with M-CSF, monocytes differentiate into MDM (Fig 1B). In all stimulated conditions three different morphological phenotypes of MDMs were observed: round, elongated, and dendritiform-like (Fig 1C–1E). Between individual donors enormous differences in cell count and morphology were observed (Fig 1F and 1G) (with the exception of donor 2 vs. donor 3, donor 2 vs. donor 6, and donor 3 vs. donor 6, all other values other comparisons differed statistically significant (p<0.001); Tukey's post hoc test). To investigate if different stimuli influence MDM morphology, round macrophages of samples stimulated either with LPS, IFNγ, or corneal tissue (FF, SF, LF) were compared to round macrophages in unstimulated samples (Fig H, at 24 hours of stimulation). Interestingly, all samples challenged with different stimuli showed a decrease in round macrophages compared to unstimulated samples (light-blue line; statistically not significant). This decrease was more pronounced in samples stimulated with corneal tissue (FF, SF, and LF in Fig 1H). The presence or absence of CECs did not influence this decrease of round macrophages.

#### Cytokine production

Cytokine production differed significantly for different stimuli and over time. Of the 34 cytokines tested, some such as IL-2, TNF-β, IL-6 (see S1 Table and S1 Fig) were not detected in media conditioned by stimulated MDM. About one-third of the cytokines screened for were highly secreted by MDM in response to incubation with human corneal tissue or the fragment-free supernatant fluid phase (Fig 2A). MDC and MCP-1 were increased in a time-dependent manner after stimulation with corneal fragments. A statistically significant upregulation of multiple cytokines over time such as MDC (p<0.001), MCP-1 (p<0.001), IL-9 (p<0.001) or IL-13 (p<0.001) was confirmed using a multifactorial linear regression model. Three chemokines, namely MCP-1, MIP-1α and MIP-1β are known to be significantly enriched in human aqueous humour during acute corneal graft rejection [11]. These were also detectable after 48 hours of MDM stimulation in the *in vitro* system (Fig 2B). Furthermore, IL-4 (p = 0.025) and IL-13 (p = 0.014), cytokines with mostly anti-inflammatory properties, were statistically significantly upregulated in the presence of corneal endothelium. In contrast, the pro-inflammatory cytokines IL-8 (p = 0.018) and MCP-3 (p = 0.016) were statistically significantly up-regulated in MDM stimulated by corneal tissue in the absence of endothelium (Fig 2C).

### Effects of MDM-conditioned media

The different cytokine patterns secreted by MDM in response to stimulation with corneal material including or excluding CEC may have different effects on target cells. To address this issue, we studied effects of MDM-conditioned media on PBMC recruitment and migration, monocyte fraction persistence and macrophage differentiation.

#### Monocyte recruitment

From a human PBMC cocktail provided, mainly monocytes migrated towards MDM-conditioned media through the transwell pores. Lymphocytes migrated to a much lesser extent compared to monocytes. While neat medium induced nearly no migration, the conditioned medium derived from MDM stimulated with corneal tissue led to an average of 50% monocytes of the total input migrating through the transwell membranes. Of these, over 80% were of the classical (“inflammatory”) type (CD14^high^/CD16^low^). The presence or absence of corneal endothelium in MDM stimulation did not significantly influence the effect of MDM-conditioned media on cell migration (Fig 3B). It is known that macrophages produce chemokines even without the presence of stimuli; this could be confirmed in our Luminex assay (see chemokine upregulation–Fig 2A). In our migration experiments the medium of unstimulated MDM induced similar levels of PBMC migration compared to the medium of MDM stimulated with corneal fragments (p = 0.7). Migration experiments using a 1:10 dilution of the MDM supernatant suggest a dose-dependent effect (see S3 Fig).

#### Monocyte fraction analysis

Monocytes have a half-life of about 19 hours in circulation [19]. Yet, viable monocytes are present in the anterior chamber during corneal graft rejection [10]. This prompted us to further investigate the survival time of monocytes in our system.

Freshly isolated monocytes from whole PBMCs were incubated in supernatant from MDM stimulated with corneae either with or without CECs. Purity of monocytes was above 95% in all samples (see FACS plots in Fig 4 and S2 Fig). Without the presence of endothelium, the monocyte content at six hours was higher and dropped to about one third after 72 h–the same was true for the lymphocyte fraction within these samples. With endothelium the monocyte content was lower at six hours, and remained at low levels at 72 hrs of incubation, whereas the lymphocyte fraction decreased within the same time-frame as seen in the samples without endothelium (Fig 4A–4E).

To further characterize monocyte subsets we used CD14 and CD16 to differentiate between classical, intermediate, and non-classical monocytes. Interestingly, monocytes incubated with supernatant from MDM challenged with endothelium showed a significant increase in intermediate monocytes. This increase in intermediate monocytes could not be detected in samples where corneal endothelial cells were absent during MDM stimulation.

#### Monocyte differentiation

Freshly isolated monocytes were incubated in unconditioned control medium or MDM-conditioned media for 72 hours. Microscopy of these monocytes revealed a macrophage-like phenotype (Fig 5A) only in cells exposed to MDM-conditioned media.

FACS analysis confirmed the presence of macrophage-like cells. Furthermore, upregulation of CD14 and MHCII as well as down-regulation of CD11b and CD86 indicated a monocyte-to-macrophage transition after 72 hours of incubation (Fig 5B and 5C).

Cells counted after incubation in cornea-stimulated MDM-conditioned medium revealed an endothelium-dependent difference: we observed a reduced monocyte-to-macrophage differentiation when corneal endothelial cells had been present in MDM stimulation (Fig 5D).

## Discussion

Most studies on cellular mechanisms of corneal graft rejection currently available rest on animal models. In a murine keratoplasty model, macrophages were found to be the first cells to markedly infiltrate the graft [20], whereas the depletion of macrophages prolonged graft survival and reduced the cellular influx in rat models [12,21,22]. In rejected human corneal specimens the predominant infiltrating cells were identified as macrophages, and to a lesser extent, T-cells [23]. To complement existing animal models, it was our goal to further elucidate possible activation mechanisms of the innate immune system in human corneal graft rejection in a well-defined setup based on human cells. Therefore, we generated a human *in vitro* model by stimulating human macrophages with human corneal tissue preparations. This allowed us to analyze human antigen detection by human macrophages, subsequent cell activation, and cytokine secretion under controlled conditions.

### Morphology and cytokines

It is known that human macrophage cell lines and murine macrophages can change their cell shape in response to different stimuli [16]. We used our *in vitro* model to investigate if human MDM change their predominant shape in response to stimulation with corneal antigen. We identified three different MDM cell phenotypes. Significant MDM donor-dependent differences in morphology distribution patterns were detected (Fig 1F). Furthermore, these patterns were altered when MDM were challenged with different stimuli. Interestingly, samples challenged with corneal tissue showed the highest decrease in round macrophages (Fig 1H) compared to MDM stimulated with LPS or IFNγ.

Nevertheless, analysis of cytokines produced by MDM in response to stimulation with corneal tissue revealed striking parallels to cytokines found in aqueous humor samples of patients with an acute corneal graft rejection, such as MCP-1, MIP-1α and MIP-1β. These three CC-chemokines specifically bind to receptors that are expressed on monocytes, namely CCR-1, -2, and -5. Amongst these receptors, CCR2 has been shown to be important for the recruitment of monocytes in the context of neuro-inflammation and breakdown of the blood-brain barrier [24].

The chemokine system shows a high redundancy in terms of ligands and receptors, suggesting that a combination of chemokines, rather than a single one, induces monocyte recruitment (Fig 3). The pattern of chemokines upregulated in our *in vitro* samples resembles earlier observations in aqueous humor [11] and seems to be responsible for the recruitment of monocytes [25], especially into the anterior chamber. Furthermore, not just the combinations of these chemokines, but also the fold-increases in our in vitro samples reproduce a pattern that can be detected in human AC samples from rejection patients. These findings strongly suggest that the *in vitro* model mirrors the AC milieu at early clinical stages of human corneal graft rejection.

Nevertheless, the redundancy in the chemokine system could hamper further investigations in this field. It has to be considered that targeting of chemokine receptors will be more promising than targeting a single ligand.

Furthermore, the biology of macrophages in this context will need deeper understanding. These cells appear as a major source of cyto- and chemokines which can be found in the AC during graft rejection. Our experiments strongly suggest that these cells secrete various soluble mediators even without distinct stimulation (Fig 2) and that these mediators are potent enough to induce monocyte recruitment in a transwell migration model (Fig 3B).

### Immunogenicity of corneal endothelium

Multiple cytokines were highly expressed by MDM subjected to corneal tissue fragments or fragment-free supernatant, with distinct response patterns in the presence or absence of corneal endothelium (Fig 2C). In the presence of corneal endothelium, we detected higher levels of anti-inflammatory cytokines, while in its absence MDM produced more pro-inflammatory cytokines. Although this did not apply to all cytokines tested, these observations could indicate an immune-modulating effect of corneal endothelium on macrophages.

Investigating in the fate of cells recruited into the anterior chamber during graft rejection in a surrogate *in-vitro* model, we found that the presence of CECs significantly affected the monocyte fraction (Fig 4): the monocyte fraction is primarily smaller (at 6h) but remains constant–at the same time the lymphocyte fraction decreases. Without endothelium the initial monocyte fraction is larger and shrinks to one third at 72h. One could speculate that these differences might play a role for the outcome of graft rejection not just in terms of absolute cell counts but also in terms of selection of monocytes potentially more harmful to the endothelial cells of the graft. This idea is further supported by the interesting fact that the composition of monocyte subsets differed depending on the presence of corneal endothelium in the antigen preparations used to stimulate MDMs. The presence of corneal endothelium favored a significant increase in intermediate (CD14++CD16+) monocytes. The role of these cells has not been completely understood [26]; current studies indicate a role in local innate surveillance as well as in the pathogenesis of autoimmune diseases [27]. IL-10 has been identified as one possible inducer for intermediate monocytes in rheumatoid arthritis [28]; this cytokine is highly upregulated in supernatants from MDM stimulated with corneal tissue. Further studies will need to investigate the role of IL-10 and other endothelial cell-derived immune-modulating factors through which the corneal endothelium controls the innate immune system during graft rejection.

Immune-modulating properties of corneal endothelial cells are further suggested by the fact that monocyte-to-macrophage differentiation was reduced in media conditioned by MDM which were stimulated by tissue preparations containing corneal endothelial cells. Of these conditioned media, the one generated using fragment-free cornea supernatant for MDM stimulation had the strongest effect (Fig 5D), indicating that soluble immunosuppressive factors secreted by corneal endothelial cells may have an influence on infiltrating monocytes.

This unexpected relation of macrophage activation to endothelial-mediated immunomodulation deserves further investigation not just in terms of analyzing MDM cytokine production but also in terms of characterizing corneal endothelial cell-derived mediators involved. Complement regulatory proteins (CRPs), soluble Fas ligand (FasL) [29], and Tumor necrosis factor-related apoptosis-inducing ligand (TRAIL) [30] appear as possible corneal endothelial cell-derived candidate molecules in this context.

Thus, the corneal endothelium appears to have a complex role in the fate of corneal grafts. Its transport function maintains corneal clarity and its secretion of anti-inflammatory mediators serves to attenuate the activity of innate immune cells. At the same time, its exposition to aqueous humor renders it a prime target of host-derived leukocytes and its limited anti-inflammatory capacity may be overwhelmed once an immune response activation threshold is surmounted.

Interestingly, monocyte recruitment from a PBMC cocktail by MDM-conditioned media in a transwell migration assay was not modified by corneal endothelial contributions as mentioned above (see Fig 3).

### Clinical impact

The distinct sites of antigen uptake, processing, and presentation at the outset of corneal graft rejection in humans are still not entirely clear. In previous animal studies, corneal antigens were detected in the conjunctiva, draining lymph nodes, and even spleen and mesenteric lymph nodes [31]^,^[32,33].

Based on a wealth of data obtained in animal studies, a current concept envisions regional lymph nodes as playing a critical role in corneal transplant rejection. Antigen-presenting cells were shown to migrate to the lymph nodes and present the antigen via MHC-molecules to cells of the adaptive immune system after obtaining antigen from the graft. Thereafter, adaptive immune cells migrate from the lymph node back to the transplant and damage the corneal endothelium [34,35]. However, cornea transplant patients in early rejection rarely show enlarged regional lymph nodes in clinical practice. Our findings in a simple human *in vitro* model strongly suggest that regional phagocytic antigen processing by macrophages may indeed be sufficient to establish an inflammatory environment at the site of antigen encounter and trigger subsequent recruitment of additional innate immune cells. Thus, an early localized initiation of graft-induced innate immune responses in the anterior chamber might have the potential to launch subsequent processes leading to graft rejection without a mandatory early-phase involvement of a lymphatic drainage pathway.

Since donors of cells and corneal tissue were not identical in our experiments, allogenic effects cannot be fully excluded in this system. For further verification, PBMCs and corneal tissue from the same donor would have to be used, which is not feasible due to ethical regulations. T-cell contaminations of the MDM cultures could also affect the results, but samples were extensively washed to minimize this possibility and FACS analysis showed almost no T-cells being present in the MDM cultures.

## Conclusion

In summary, our study suggests that distinct aspects of corneal graft rejection can be modelled in a human *in vitro* system. This will allow further studies to characterize the role of human macrophages in corneal antigen uptake und subsequent immune cell-activation.

## Supporting information

S1 Table (TIF)

S2 Table (TIF)

S1 Fig (TIF)

S2 Fig (TIF)

S3 Fig (TIF)
