# Supplementary figures and images for: Activation of human macrophages by human corneal allogen in vitro
Source: PLoS One. 2018 Apr 4;13(4):e0194855. doi: 10.1371/journal.pone.0194855 (PMC5884541; doi:10.1371/journal.pone.0194855)

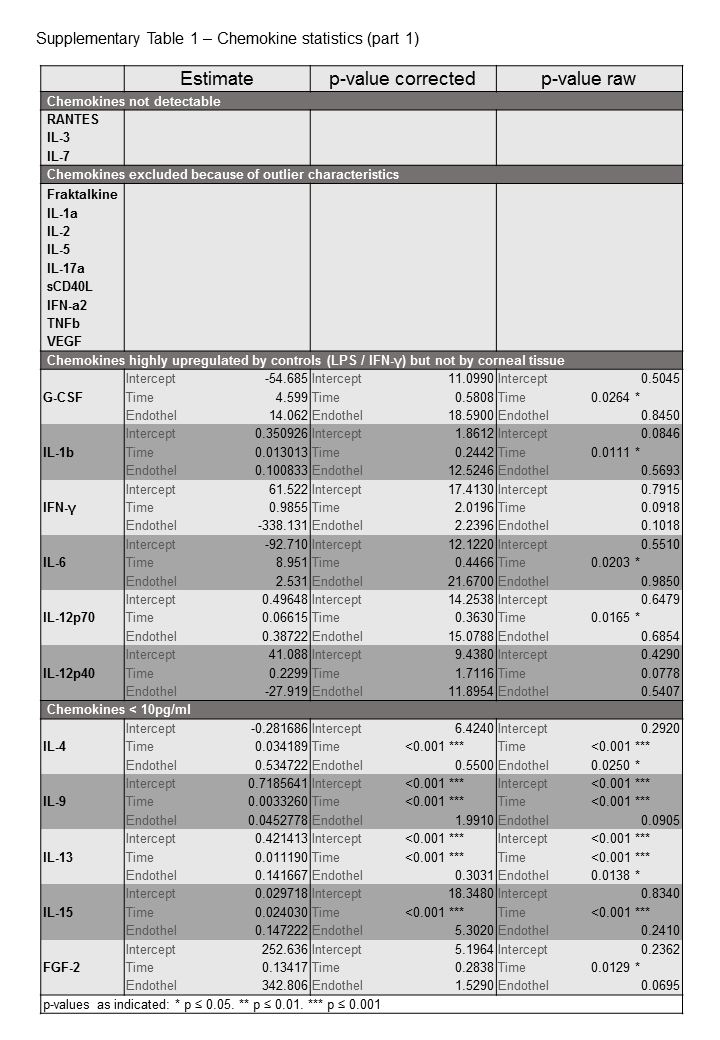

Supplement: S1 Table — (TIF) [file pone.0194855.s001.TIF]

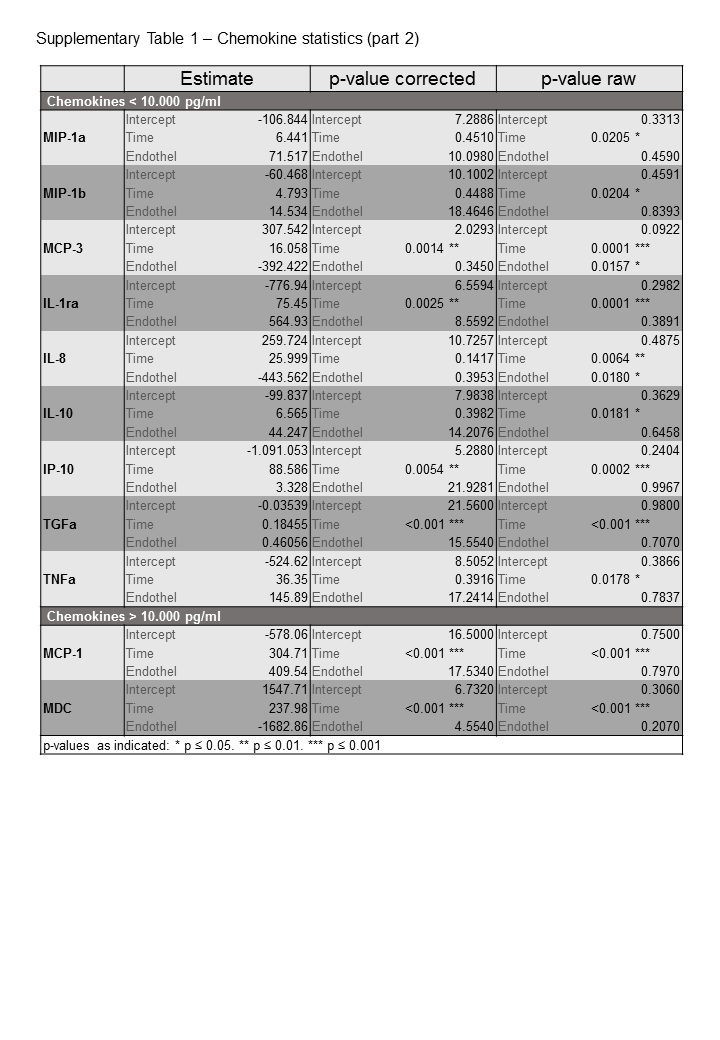

Supplement: S2 Table — (TIF) [file pone.0194855.s002.TIF]

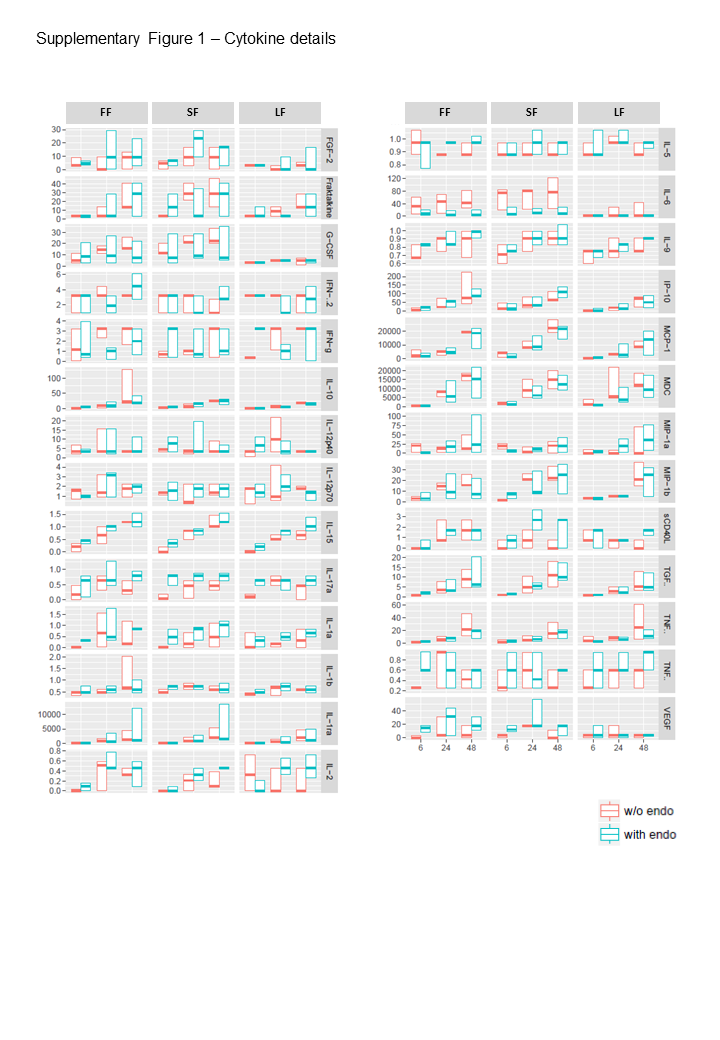

Supplement: S1 Fig — (TIF) [file pone.0194855.s003.TIF]

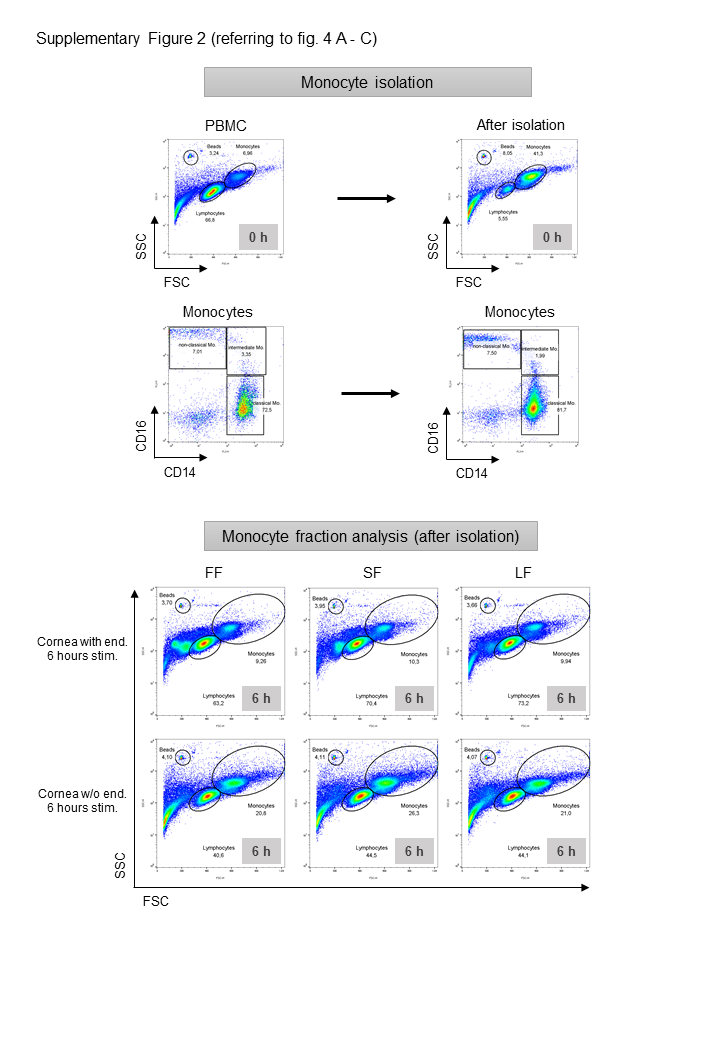

Supplement: S2 Fig — (TIF) [file pone.0194855.s004.TIF]

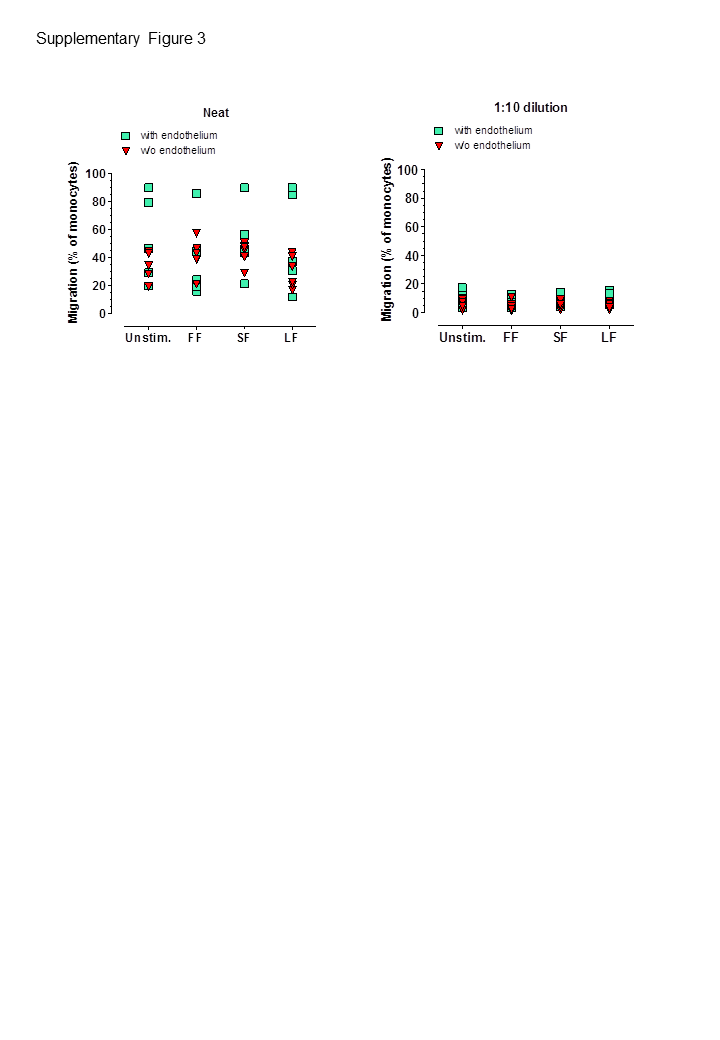

Supplement: S3 Fig — (TIF) [file pone.0194855.s005.TIF]
